# Decoding Wilson disease: a machine learning approach to predict neurological symptoms

**DOI:** 10.3389/fneur.2024.1418474

**Published:** 2024-06-19

**Authors:** Yulong Yang, Gang-Ao Wang, Shuzhen Fang, Xiang Li, Yufeng Ding, Yuqi Song, Wei He, Zhihong Rao, Ke Diao, Xiaolei Zhu, Wenming Yang

**Affiliations:** ^1^Department of Neurology, The First Affiliated Hospital of Anhui University of Traditional Chinese Medicine, Hefei, Anhui, China; ^2^School of Information and Artificial Intelligence, Anhui Agricultural University, Hefei, Anhui, China; ^3^Center for Xin'an Medicine and Modernization of Traditional Chinese Medicine, Institute of Health and Medicine Hefei Comprehensive National Science Center, Hefei, Anhui, China; ^4^Key Laboratory of Xin'An Medicine, Ministry of Education, Hefei, Anhui, China

**Keywords:** Wilson disease, machine learning, neurological symptom, prediction model, eXtreme Gradient Boosting (XGB)

## Abstract

**Objectives:**

Wilson disease (WD) is a rare autosomal recessive disorder caused by a mutation in the *ATP7B* gene. Neurological symptoms are one of the most common symptoms of WD. This study aims to construct a model that can predict the occurrence of neurological symptoms by combining clinical multidimensional indicators with machine learning methods.

**Methods:**

The study population consisted of WD patients who received treatment at the First Affiliated Hospital of Anhui University of Traditional Chinese Medicine from July 2021 to September 2023 and had a Leipzig score ≥ 4 points. Indicators such as general clinical information, imaging, blood and urine tests, and clinical scale measurements were collected from patients, and machine learning methods were employed to construct a prediction model for neurological symptoms. Additionally, the SHAP method was utilized to analyze clinical information to determine which indicators are associated with neurological symptoms.

**Results:**

In this study, 185 patients with WD (of whom 163 had neurological symptoms) were analyzed. It was found that using the eXtreme Gradient Boosting (XGB) to predict achieved good performance, with an MCC value of 0.556, ACC value of 0.929, AUROC value of 0.835, and AUPRC value of 0.975. Brainstem damage, blood creatinine (Cr), age, indirect bilirubin (IBIL), and ceruloplasmin (CP) were the top five important predictors. Meanwhile, the presence of brainstem damage and the higher the values of Cr, Age, and IBIL, the more likely neurological symptoms were to occur, while the lower the CP value, the more likely neurological symptoms were to occur.

**Conclusions:**

To sum up, the prediction model constructed using machine learning methods to predict WD cirrhosis has high accuracy. The most important indicators in the prediction model were brainstem damage, Cr, age, IBIL, and CP. It provides assistance for clinical decision-making.

## Introduction

Wilson disease (WD), alsoknown as hepatolenticular degeneration (HLD), was described by Wilson in 1912 and is a rare autosomal recessive inherited disorder (1, 2). The disease is caused by homozygous or compound heterozygous mutations in ATP7B, which encodes the transmembrane copper-transporting ATPase 2 (commonly referred to as ATP7B). Abnormal function of ATP7B can lead to impaired copper homeostasis and copper overload in the brain, liver and other organs (3). Neurological symptoms are one of the most common types of symptoms associated with this disease. According to research reports, 18–68% of WD patients experience neurological symptoms for the first time, with an average age of 20–30 years (4, 5). However, most patients exhibit liver symptoms in the early stages, followed by severe neurological symptoms (1). Although UWDRS, GAS and other scales can assess the neurological symptoms of patients, the treatment effect is often poor when patients experience neurological symptoms (6–8). Therefore, predicting and intervening in advance on the relevant influencing factors that cause neurological symptoms in patients is crucial for their prognosis.

Machine learning is a research field dedicated to understanding how computers acquire knowledge from data (9). Machine learning technology involves computer-based statistical methods that are trained to identify common patterns and hidden correlations in large datasets. In clinical practice, machine learning methods can help clinicians classify patients with multiple diseases according to several variables (10–12). In recent years, multiple studies have been reported on the application of machine learning methods in predicting Parkinson's disease and Alzheimer's disease (13, 14). In addition, research on machine learning-based prediction of liver cirrhosis in WD has also achieved great success (15). However, so far, no model was used to predict the development of neurological symptoms in WD.

**Figure 1:**
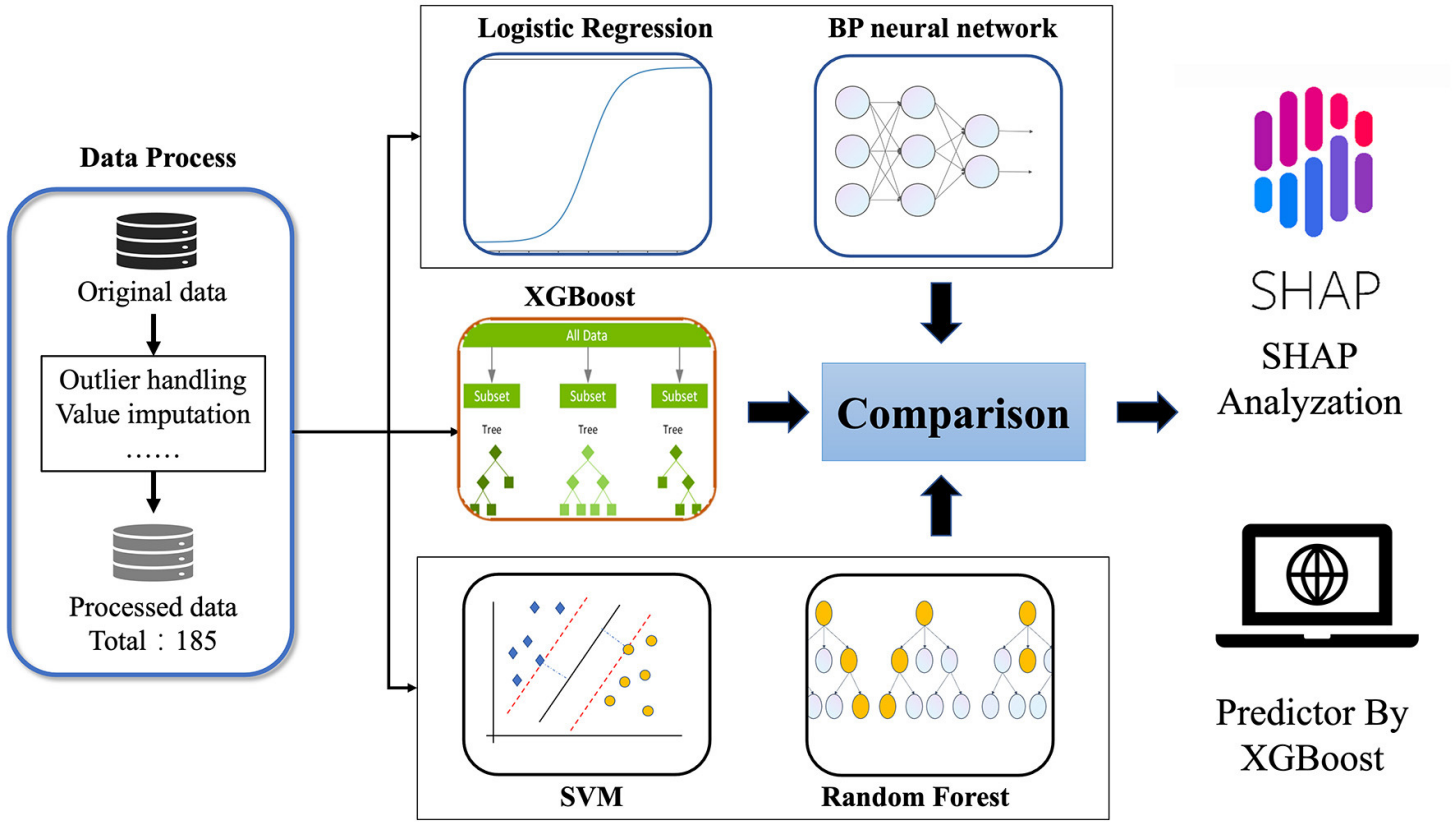
The flowchart of this study.

Therefore, this study combines machine learning with clinical multidimensional indicators, such as general clinical information, imaging examinations, blood and urine tests, and clinical scale measurements, to determine the most important indicators related to WD neurological symptoms and construct a predictive model for the occurrence of neurological symptoms in WD patients. Intended to provide clinical decision-making tools for clinicians, it is hoped that through early intervention of these important factors, patients can delay or even avoid the occurrence of neurological symptoms. The flowchart of this study was shown in Figure 1.

## Materials and methods

### Study design and population

The study population included 185 WD patients enrolled between July 2021 and October 2023. All patients were inpatients of the Brain Disease Center of the First Affiliated Hospital of Anhui University of Traditional Chinese Medicine and conformed to the EASL clinical practice guidelines for WD (16). The inclusion criteria were as follows: age 12–60 years old (Patients < 12 years old may not be able to cooperate in completing MRI and other examination, while patients >60 years old may exhibit symptoms that are confused with the symptoms of this disease due to age); routine copper depletion treatment at a stable dosage in the previous 4 weeks. The exclusion criteria were as follows: age < 12 years old or >60 years old; unable to cooperate in completing the scale assessment; combined with other neurological, orthopedic, or cardiovascular/respiratory diseases; combined with severe mental symptoms.

This study was conducted following the 1964 Declaration of Helsinki and approved by the Ethics Committee of the First Affiliated Hospital of Anhui University of Traditional Chinese Medicine (Approval No.: 2021AH-66). All subjects received written informed consent.

### Data processing

In this study, we included data from 185 patients for prediction (please refer to Supplementary material for details). Before conducting data prediction, outliers were discarded, and for the discarded outliers and missing values, we used a imputation method. If the discarded outliers and missing values are of discrete type, we use mode imputation. If the discarded outliers and missing values are of continuous type, we use mean to randomly select imputation within the variance range. To validate the reliability of the imputed data, we compared the means, medians, and standard deviations before and after imputation, with errors within 3%. Subsequently, we conducted machine learning cross-validation, further confirming the reliability of the imputed data. Meanwhile, the R code was implemented in Empower Stats. Based on previous research results, eligible WD patients were randomly divided into a training group and a testing group at a ratio of 9:1 (17, 18). For some discrete-valued features such as the presence of cirrhosis and ascites, the plurality complement was used, and for some continuous-valued features, the mean complement method was used. Modeling was performed on the training set, followed by validation on an independent test set. The data is presented in Table 1.

**Table 1:** Number of data set.

	**Training set**	**Independent test set**	**Total**
Positive	149	14	163
Negative	16	6	22

### Neuropsychiatric assessment and clinical objective indicators

All subjects were assessed for neuropsychiatric symptoms using the Unified Wilson Disease Rating Scale (UWDRS) (8). The full UWDRS includes three subscales: the neurological subscale, the liver subscale, and the psychiatric subscale. Since this experiment was mainly to evaluate the neurological symptoms of patients, the subjects were classified into those with neurological symptoms (WD-N, *n* = 163) and those without neurological symptoms (no-WD-N, *n* = 22) according to whether the score on the neurological subscale was zero or not. Additionally, through the electronic medical record system, this study collected the following information as predictive factors: gender, age, blood routine examination, liver function, kidney function, liver fibrosis, blood clotting function, 24 h urinary copper, ceruloplasmin, cranial magnetic resonance lesion sites, abnormal liver ultrasound findings and the presence of K-F rings, as well as cirrhosis and ascites.

### Statistical analysis

IBM SPSS 25.0 statistical software was utilized to compare the characteristics of demographics, clinical objective indicators, and scale measures of WD patients with and without neurological symptoms. Continuous variable data were expressed as mean ± SD, and differences between groups were analyzed using *t-*test. Meanwhile, categorical variable data were expressed as numbers and percentages, and the difference between groups was analyzed using the Chi-squared test or Fisher's exact test. Specifically, *P* > >0.05 indicates that the statistical results have no statistically significant difference, *P* < 0.05 indicates a statistically significant difference, *P* < 0.01 indicates a highly statistically significant difference, and *P* < 0.001 indicates an extremely statistically significant difference.

### Machine learning methods

Five common machine learning methods, namely Random Forest (RF), Support Vector Machine (SVM), eXtreme Gradient Boosting (XGB), Logistic regression (LR), and Back propagation (BP) neural network were used to model the data in this study (19–23).

**RF**: Random forest (19) is a machine learning method based on integrated learning of decision trees. It adopts the concepts of bootstrap sampling and feature randomization to construct multiple decision trees for prediction. Then, it aggregates the results of multiple decision trees through voting to improve prediction accuracy and robustness.

**SVM**: SVM can be used for classification tasks, serving as a supervised learning algorithm (21). For binary classification problems, the objective of SVM is to find a hyperplane that maximizes the margins between different classes.

**XGB**: XGB (20) is a machine learning algorithm implemented in the gradient boosting framework. It is an improved algorithm based on gradient boosting machines with the incorporation of a regularization term to effectively prevent the overfitting problem. It has been widely used in the field of machine learning owing to its high prediction accuracy and computational efficiency.

LR: LR (22) is a statistical model used for binary classification tasks, where the goal is to predict the probability that an instance belongs to a particular class. Unlike linear regression, which predicts a continuous value, logistic regression predicts the probability of the instance belonging to the default class (class 1 in binary classification) using a logistic function (specific algorithms and parameters can be found in the Supplementary material).

BP: BP neural network (23), often simply referred to as neural networks, are a type of artificial neural network that is widely used for supervised learning tasks, including classification and regression. They are composed of multiple layers of interconnected nodes, or neurons, organized in a hierarchical manner (specific algorithms and parameters can be found in the Supplementary material).

**Shapley Additive Explanations:** The Shapley Additive Explanations (SHAP) algorithm (24) is a unified framework for explaining predictions of black-box models. It assigns importance scores to each feature to determine the contribution of each feature to the final model prediction. Different from the XGB model which only computes feature importance, the SHAP feature selection method evaluates the impact of each feature not only by calculating its SHAP value but also by explaining the specific behavior of the feature that affects the prediction results. The SHAP algorithm has been increasingly used as a model interpretation method in fields such as biomedical research. The calculation of SHAP values is given below:


$$\begin{array}{c} \phi_{i}=\underset{S\subseteq \left\{1,2,\ldots ,p\right\}\backslash \left\{i\right\}}{\sum }\frac{|S|!\left(p-|S|-1\right)!}{p!}\left[f\left(S\cup \left\{i\right\}\right)-f\left(S\right)\right] \end{array}$$

where ϕ_*i*_ represents the SHAP value for feature *i*, *p* denotes the number of features, S denotes a subset composed of features other than feature *i*, |*S*| denotes the size of the set *S*, and *f*(*S*) denotes the model prediction output for input feature set *S*. If the SHAP value ϕ_*i*_ is greater than zero, it is considered that the feature positively contributes to the model prediction. Conversely, if the SHAP value is less than zero, it indicates that the feature negatively affects the model prediction.

The experimental environment utilized Python 3.8.12, with RF and SVM constructed using the sklearn 1.2.1 package. The XGB package version employed was 1.5.0, and the shap package version used was 0.39.0.

### Model evaluation metrics

To evaluate the performance of the model and facilitate a more intuitive comparison with other methods, this study took the area under the receiver operating characteristic (AUROC) curve and the area under the precision-recall curve (AUPRC) as the primary evaluation metrics. The receiver operating characteristic (ROC) curve is a vital measure of the model's robustness. Given the imbalanced nature of the independent test datasets used in this study, the area under the precision-recall curve (AUPRC) was also used for model evaluation. Additionally, specificity (SP), sensitivity (SN), accuracy (ACC), and Matthew's correlation coefficient (MCC) were chosen as additional evaluation parameters, and their definitions are as follows:


$$\begin{array}{c} \begin{array}{c} Sn=\frac{TP}{TP\;+\;FN} \end{array} \end{array}$$

$$\begin{array}{c} \begin{array}{c} Sp=\frac{TN}{TN\;+\;FP} \end{array} \end{array}$$

$$\begin{array}{c} \begin{array}{c} ACC=\frac{TP\;+\;TN}{TP\;+\;TN\;+\;FP\;+\;FN} \end{array} \end{array}$$

$$\begin{array}{c} \begin{array}{c} MCC=\frac{TP\times TN\;-\;FP\times FN}{\sqrt{\left(TP\;+\;FP\right)\left(TP\;+\;FN\right)\left(TN\;+\;FP\right)\left(TN\;+\;FN\right)}} \end{array} \end{array}$$

where TP, TN, FP, and FN represent true positives, true negatives, false positives, and false negatives, respectively.

## Results

### Comparison of characteristics of patients with WD

There was no statistically significant difference between the WD-N group and the no-WD-N group in terms of gender, presence or absence of cirrhosis, ascites, cerebral cortex damage, liver capsule smoothing, enhanced internal echo in the liver, homogeneous echogenicity in the liver, and clear internal blood vessels in the liver (*P* > 0.05). Meanwhile, there was no statistical difference between the WD-N group and the no-WD-N group in terms of WBC, RBC, Hb, PLT, PT, INR, APTT, FBG, TT, ALT, AST, TBA, TBIL, DBIL, IBIL, TP, ALB, GGT, ALP, BUN, and 24-h urine copper (*P* > 0.05). Additionally, there was a statistically significant difference between the WD-N group and the no-WD-N group in terms of K-F ring, lenticular nucleus damage, thalamus damage, brainstem damage, cerebral ventricular system dilation, deepening of the sulci and fissures of the brain (*P* < 0.001), cerebral peduncle damage, and other brainstem damage (*P* < 0.05). Furthermore, the age (*P* < 0.001), Cr, psychiatric symptom scores, and liver symptom scores (*P* < 0.01) of the WD-N group were significantly higher than those of the no-WD-N group, while CP (*P* < 0.05) was significantly lower than that of the no-WD-N group. The detailed information and the units of all the variables are listed in Table 2.

**Table 2:** Subtype information of WD patients at baseline and follow-up.

	**WD-N**	**no-WD-N**	***P***
N	163	22	
Gender (Male/Female)	105/58	12-Oct	0.1028
K-F ring (Yes/No)	158/5	14-Aug	< 0.001^***^
Cirrhosis (Yes/No)	100/63	12-Oct	0.1708
Ascites (Yes/No)	5/158	21-Jan	0.5374
Lenticular nucleus damage (Yes/No)	144/19	14-Aug	< 0.001^***^
Thalamus damage (Yes/No)	98/65	19-Mar	< 0.001^***^
Brainstem damage (Yes/No)	121/42	18-Apr	< 0.001^***^
Cerebral ventricular system dilation (Yes/No)	122/41	15-Jul	< 0.001^***^
Deepening of the sulci and fissures of the brain (Yes/No)	121/42	16-Jun	< 0.001^***^
Cerebral peduncle damage (Yes/No)	73/90	20-Feb	0.0010^**^
Cerebral cortex damage (Yes/No)	24/139	21-Jan	0.3185
Other brain regions damage (Yes/No)	70/93	18-Apr	0.0354^*^
Liver capsule smoothing (Yes/No)	120/43	17/5	0.8015
Enhanced internal echo in the liver (Yes/No)	163/0	21/1	0.1189
Homogeneous echogenicity in the liver (Yes/No)	163/0	21/1	0.1189
Clear internal blood vessels in the liver (Yes/No)	119/44	18/4	0.4481
Age (year)	28.33 ± 8.53	21.77 ± 6.52	< 0.001^***^
WBC (10∧9/L)	4.78 ± 1.73	5.53 ± 1.76	0.0605
RBC (10∧12/L)	4.38 ± 0.52	4.33 ± 0.48	0.6772
Hb (g/L)	128.81 ± 19.33	129.00 ± 15.28	0.9646
PLT (10∧9/L)	154.94 ± 76.28	174.91 ± 84.98	0.2604
PT (s)	11.30 ± 1.52	11.18 ± 0.82	0.7117
INR	1.01 ± 0.10	0.99 ± 0.08	0.2237
APTT (s)	30.87 ± 3.29	30.15 ± 2.98	0.3405
FBG (g/L)	2.42 ± 0.57	2.15 ± 0.33	0.4925
TT (s)	17.70 ± 2.59	17.68 ± 0.98	0.9613
ALT (U/L)	30.68 ± 26.15	40.07 ± 33.15	0.1318
AST (U/L)	28.14 ± 15.02	27.15 ± 11.10	0.7663
TBA (μmol/L)	10.34 ± 12.06	8.26 ± 9.42	0.4399
TBIL (μmol/L)	16.18 ± 7.09	15.28 ± 7.46	0.5845
DBIL (μmol/L)	3.35 ± 2.24	2.98 ± 1.27	0.4556
IBIL (μmol/L)	12.84 ± 5.36	12.30 ± 6.40	0.6709
TP (g/L)	63.18 ± 5.95	63.00 ± 3.90	0.8858
ALB (g/L)	40.55 ± 15.63	38.34 ± 2.54	0.5116
GGT (U/L)	41.87 ± 42.93	38.64 ± 30.31	0.7347
ALP (U/L)	110.48 ± 65.74	106.41 ± 40.80	0.7787
BUN (mmol/L)	5.21 ± 5.01	4.20 ± 1.14	0.3514
Cr (μmol/L)	70.02 ± 16.62	59.49 ± 11.11	0.0046^**^
24-h urine copper (μg/24h)	871.50 ± 758.29	918.95 ± 591.11	0.7796
CP (g/L)	0.04 ± 0.03	0.05 ± 0.05	0.0439
Psychiatric symptom scores	4.76 ± 4.06	1.77 ± 2.84	0.0011^**^
Liver symptom scores	1.78 ± 1.67	0.77 ± 0.85	0.0064^**^

N, number; K-F ring, Kayser-Fleischer ring; WBC, white blood cell; RBC, red blood cell; Hb, hemoglobin; PLT, platelet count; PT, prothrombin time; INR, international normalized ratio; APTT, activated partial thromboplastin time; FBG, fibrinogen; TT, thrombin time; ALT, alanine aminotransferase; AST, aspartate aminotransferase; TBA, total bile acid; TBIL, total bilirubin; DBIL, direct bilirubin; IBIL, indirect bilirubin; TP, total protein; ALB, albumin; GGT, γ-Glutamyl Transferase; ALP, alkaline phosphatase; BUN, blood urea nitrogen; Cr, creatinine; CP, ceruloplasmin; ^*^, *P* < 0.05; ^**^, *P* < 0.01; ^***^, *P* < 0.001.

### Selection of machine learning modeling methods

**Figure 2:**
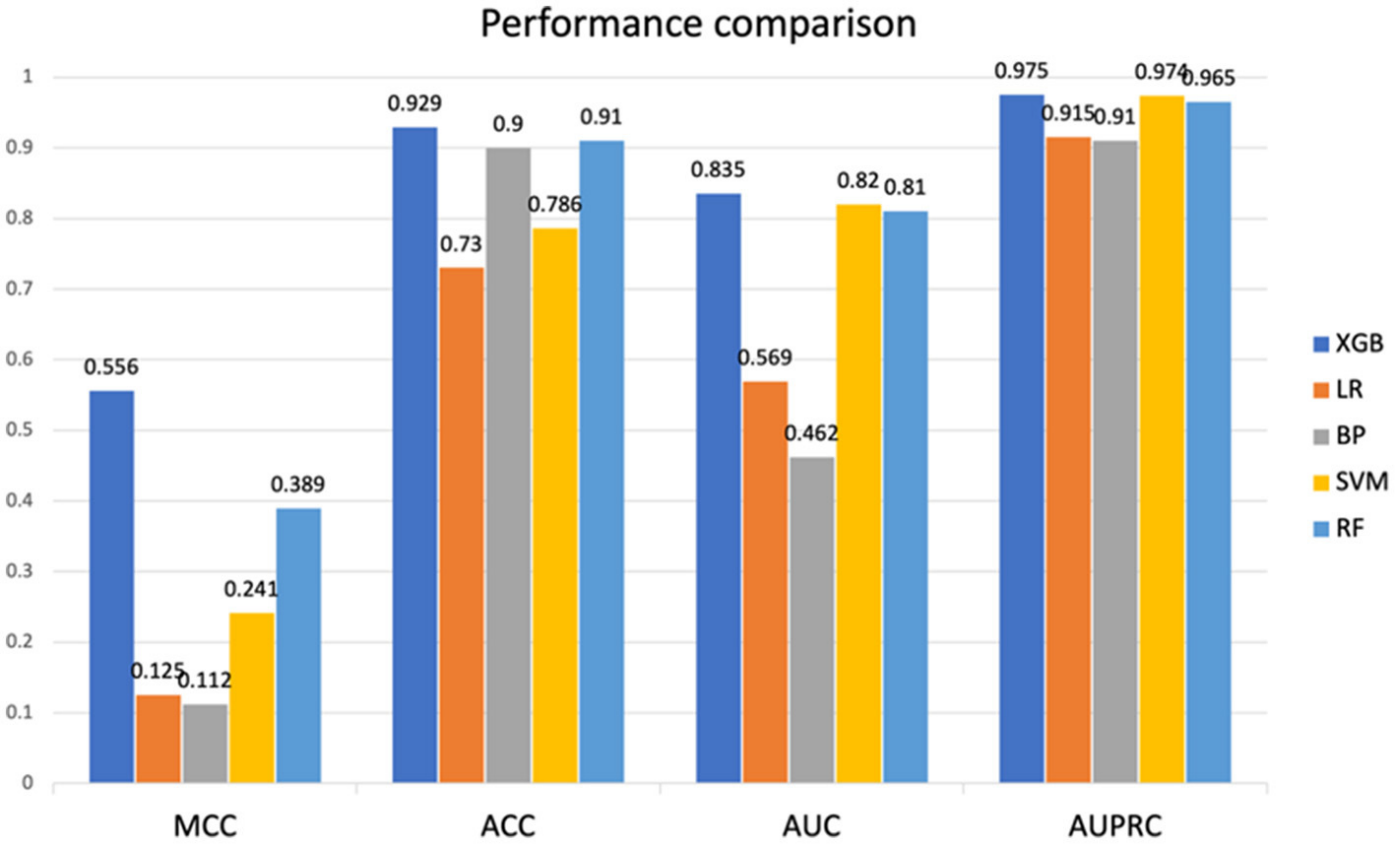
Performance comparison between SVM, RF, XGB, LR, and BP on test sets. SVM, Support Vector Machine; RF, Random Forest; XGB, eXtreme Gradient Boosting; LR, logistic regression; BP, back propagation neural network. MCC, Matthews correlation coefficient; ACC, accuracy; AUC, area under the receiver operating characteristic curve; AUPRC, area under the precision-recall curve.

**Figure 3:**
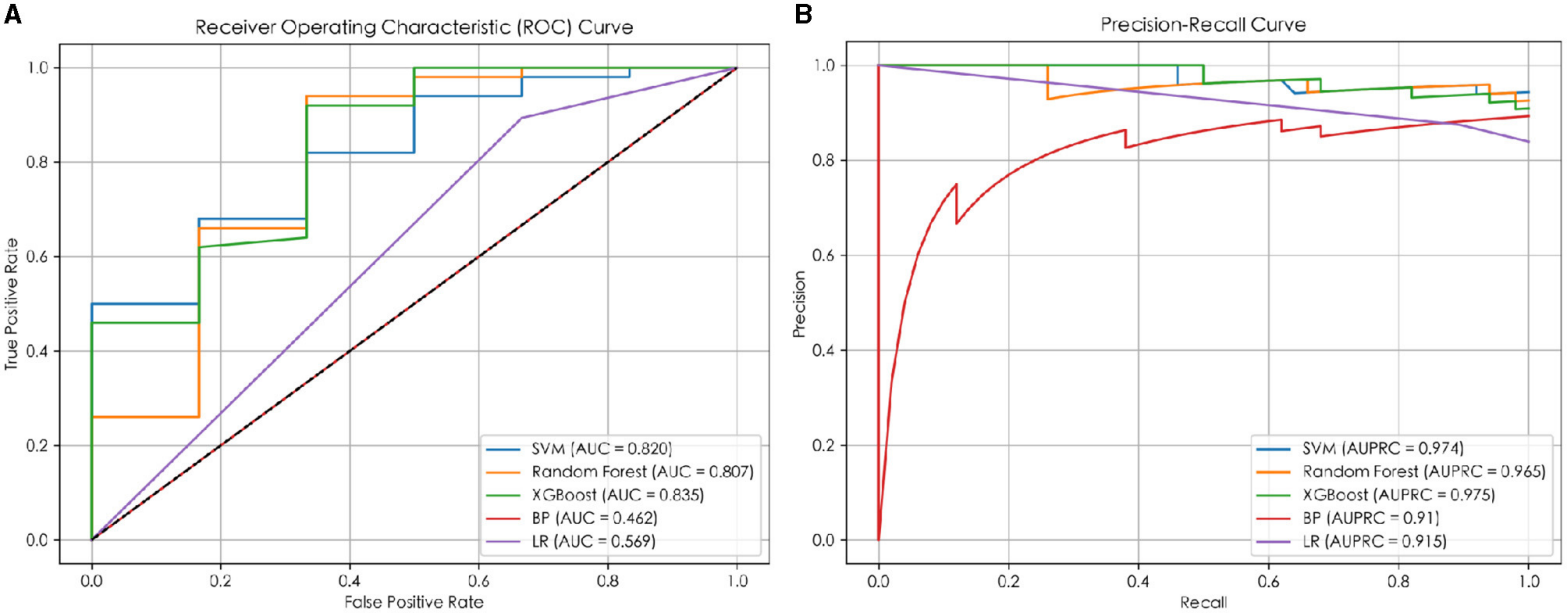
The ROC and PR curves of the prediction models. **(A)** The ROC curve of the prediction model; **(B)** the PR curve of the prediction model.

In this study, five machine learning methods, namely XGB, RF, SVM, LR, and BP neural network, were used to construct a prediction model. Firstly, the training set was used for modeling through 5-fold cross-validation (25) and grid search (26). Then, the optimal hyperparameters were obtained. Finally, the model was tested on an independent test set using a selection of this model. The results indicated that the XGB model achieved the best prediction performance among the five machine learning models, and the MCC, ACC, AUC, and AUPRC values of the XGB model on the test set were 0.556, 0.929, 0.835, and 0.975 respectively (details are provided in Figure 2), and the AUC and AUPRC curves of the XGB model were plotted (the details are provided in Figures 3A, B).

As shown in the Figure 2, compared to XGB, LR, and BP neural network exhibit significant differences in test results. This is because the data we used are non-linear and discrete, with relatively small feature dimensions, making this feature type very suitable for algorithms developed based on tree models. Similarly, the results of XGB and RF based on tree models mentioned earlier also differ from SVM, as described in the text above.

### Summary of predictive factors for neurological symptoms prediction models

**Figure 4:**
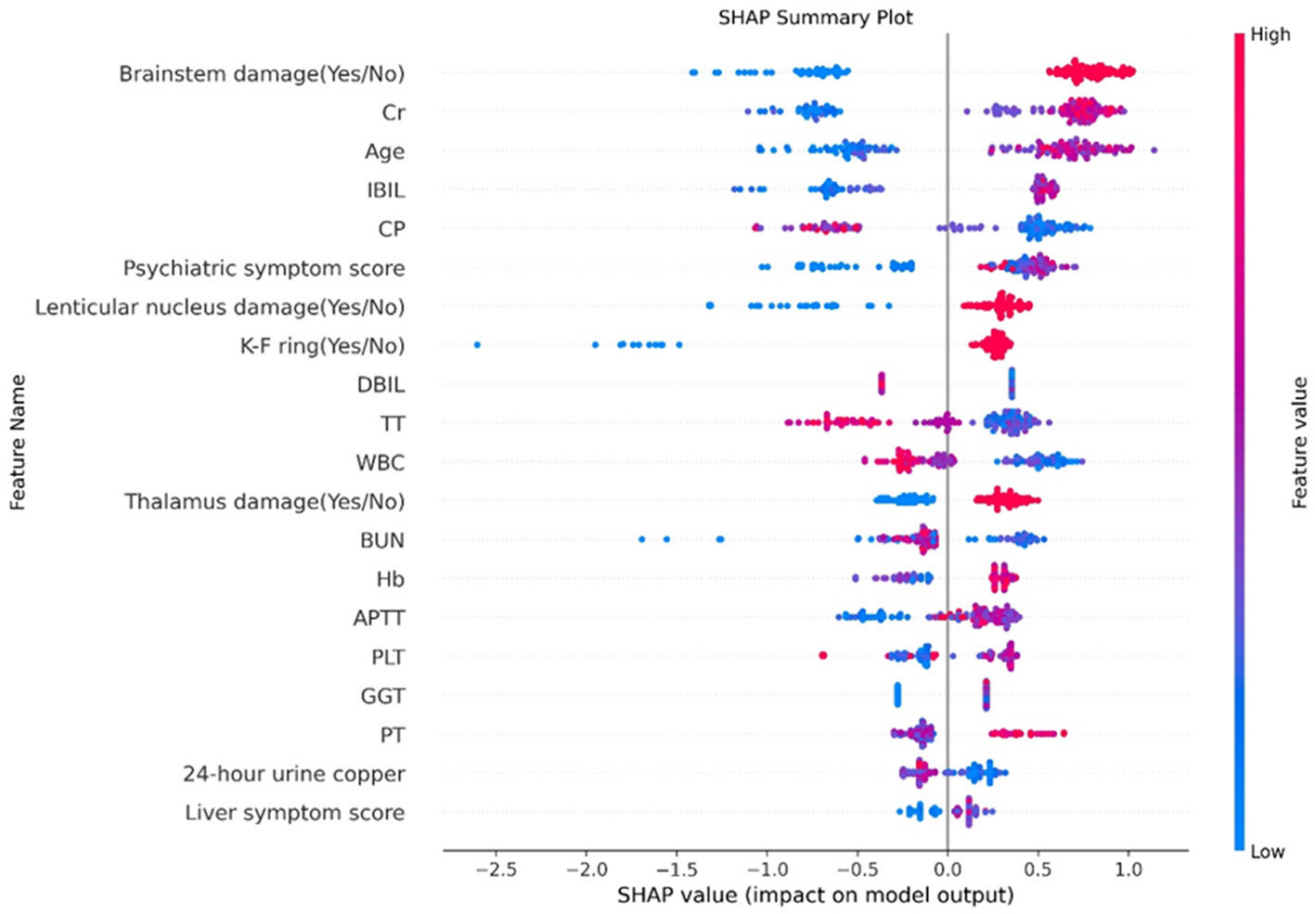
Feature analysis results. The abscissa represents the weight of the influence, instead of the specific value of the feature; the influence of feature value size on the results is represented by color (red represents a large value, blue represents a small value, and purple is adjacent to the mean). Cr, creatinine; IBIL, indirect bilirubin; CP, ceruloplasmin; DBIL, direct bilirubin; TT, thrombin time; WBC, white blood cell; BUN, blood urea nitrogen; Hb, hemoglobin; APTT, activated partial thromboplastin time; PLT, platelet count; GGT, γ-Glutamyl Transferase; PT, prothrombin time.

**Figure 5:**
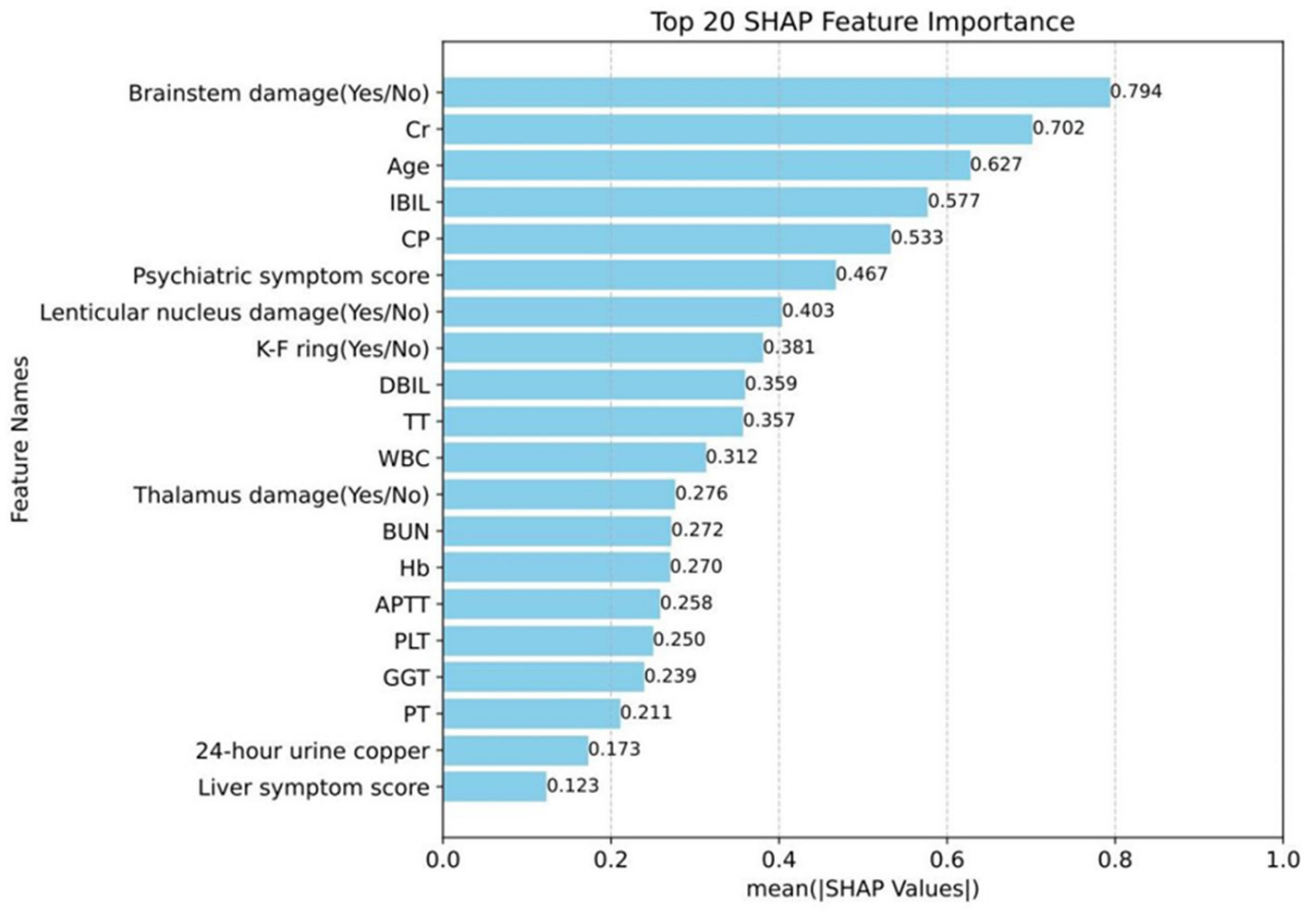
Magnitude of the influence of the indicator on neurological symptoms. Cr, creatinine; IBIL, indirect bilirubin; CP, ceruloplasmin; DBIL, direct bilirubin; TT, thrombin time; WBC, white blood cell; BUN, blood urea nitrogen; Hb, hemoglobin; APTT, activated partial thromboplastin time; PLT, platelet count; GGT, γ-Glutamyl Transferase; PT, prothrombin time.

Additionally, the SHAP model interpretation package was used to analyze the features of the XGB classifier and graph all instances so that the magnitude of the feature's influence on the prediction can be observed. The wider the regional distribution, the greater its influence (the details are illustrated in Figure 4). Then, to plot a standard bar graph representing the significance of each feature, the average absolute values of the SHAP values were calculated for each feature (the details are illustrated in Figure 5). Based on the Gini impurity, it was found that the most important predictors in the prediction model were brainstem damage, Cr, age, IBIL, and CP.

## Discussion

As far as we know, this is the first prospective case-control study to combine machine learning methods with multidimensional clinical data to predict the onset of neurological symptoms in WD patients. In this study, the XGB method in machine learning was employed to construct models to predict the occurrence of neurological symptoms in WD patients. The MCC, ACC, AUC, and AUPRC values on the test set were 0.556, 0.929, 0.835, and 0.975, respectively, achieving good results. The results suggested that brainstem damage (0.794), Cr (0.702), age (0.627), IBIL (0.577), and CP (0.533) were the top five important predictors of Gini impurity, and the presence of brainstem damage and the higher the values of Cr, Age, and IBIL, the more likely neurological symptoms were to occur, while the lower the CP value, the more likely neurological symptoms were to occur.

When comparing the demographic and clinical characteristics of the patients in the WD-N and no-WD-N groups, the latter were significantly older than the former, which is consistent with previous findings (27). Meanwhile, serological tests showed that patients with neurological symptoms had a significantly higher Cr value and a significantly lower CP value than those without neurological symptoms, and both were good predictors of the occurrence of neurological symptoms. Previous studies have found that Cr may have a certain correlation with neurological symptoms (28). Additionally, previous studies have found that CP plays an important role in the metabolism and development of neural tissues (29), and the deficiency or functional impairment of ceruloplasmin is a typical feature of various neurodegenerative diseases (30, 31). Therefore, it can be speculated that elevated Cr with diminished CP may be associated with the development of neurological symptoms. Moreover, it was found that the WD-N group had a higher incidence of the K-F ring than the no-WD-N group. These results are consistent with previous research findings (16, 32–34). Furthermore, the WD-N group had significantly higher psychiatric symptom scores and liver symptom scores than the no-WD-N group. This suggests that neurological patients often have psychiatric symptoms, and they tend to have more pronounced hepatic symptoms than hepatic patients. Also, it was found that the WD-N group had a higher incidence of lenticular nucleus damage, thalamus damage, brainstem damage, cerebral ventricular system dilation, deepening of the sulci, and fissures of the brain cerebral peduncle damage and other brain region damage than the no-WD-N group. This finding is also consistent with clinical practice. However, some patients have imaging manifestations but no neurological symptoms in clinical practice, and the accuracy of determining whether a patient has neurological symptoms by looking at them for imaging damage is not very high, which has drawn our attention.

Therefore, this study employed a machine learning approach to predict the onset of neurological symptoms in WD patients, in an attempt to find more sensitive predictive indicators. In this experiment, five machine learning methods, namely RF, SVM, XGB, logistic regression, and BP neural network, were used to construct a prediction model, and the results indicated that the XGB model achieved the best prediction performance among the five machine learning models. Finally, the SHAP model interpretation package was utilized to analyze the features of the XGB classifier. It was found that the most important predictors in the prediction model were brainstem damage, Cr, age, IBIL, and CP. The brainstem or truncus cerebri forms the intermediate part of the vertebrate central nervous system, and it is rostral to the diencephalon and caudal to the spinal cord (35). Brainstem damage is correlated with multiple neurological symptoms of ataxia, dysarthria, gait abnormalities, and muscle spasms (36–38). Additionally, age is also an important predictor of neurological symptoms in WD patients. Considering the previous findings that the average age of N-WD is older than that of no-N-WD (27), it can be speculated that the likelihood of neurological symptoms in WD patients increases with age. A study has found that the bilirubin/albumin ratio is an important risk factor for the occurrence of hepatic encephalopathy, and reducing free bilirubin may provide a new approach to the treatment of hepatic encephalopathy. It can be seen that indirect bilirubin is also closely related to neurological symptoms (39). Finally, it was found that CP is an important predictor of WD. Combined with our finding that CP values in the N-WD group were lower than those in the no-N-WD group, it can be speculated that the lower the CP, the more likely patients are to develop neurological symptoms.

To sum up, the presence of brainstem damage and the higher the values of Cr, Age, and IBIL, the more likely neurological symptoms were to occur, while the lower the CP value, the more likely neurological symptoms were to occur. Therefore, when the patient is older, it is important to be alert to the occurrence of neurological symptoms; When patients experience brainstem damage or low CP values, we should actively intervene to prevent neurological symptoms from occurring. During daily care, patients should be advised to reduce their intake of foods that can cause elevated Cr and IBIL levels, and actively reduce Cr and IBIL when it is high to prevent the occurrence of neurological symptoms.

However, our study still has some limitations. Firstly, the study does not include external validation, which increases the risk of overfitting. Secondly, the number of samples in this study queue does not match well, so there exists selection bias and recall bias. Finally, this prediction model can only predict the presence of neurological symptoms and cannot evaluate the severity of neurological symptoms. Therefore, our future work aims to address the issues of data scarcity and imbalance by using methods such as oversampling or data augmentation.

## Conclusions

In conclusion, the prediction model for predicting neurological symptoms in WD, constructed by using the XGB method in machine learning, achieved good performance, and furthermore, we will continue to collect patient data to keep the model updated. Meanwhile, the SHAP method was used to analyze clinical indicators and their impact on neurological symptoms. The results revealed that the most important factors in the prediction model were brainstem damage, Cr, age, IBIL, and CP. And the presence of brainstem damage and the higher the values of Cr, Age, and IBIL, the more likely neurological symptoms were to occur, while the lower the CP value, the more likely neurological symptoms were to occur. It provides assistance for clinical decision-making.

## Data availability statement

The training data presented in the study is included in the article/Supplementary material, further inquiries can be directed to the corresponding authors.

## Ethics statement

The studies involving humans were approved by the First Affiliated Hospital of Anhui University of Chinese Medicine (Approval number: 2021AH-66). The studies were conducted in accordance with the local legislation and institutional requirements. Written informed consent for participation in this study was provided by the participants' legal guardians/next of kin.

## Author contributions

YY: Writing – original draft, Writing – review & editing, Data curation, Formal analysis, Methodology, Project administration, Resources, Supervision, Validation. G-AW: Writing – original draft, Writing – review & editing, Data curation, Methodology, Supervision. SF: Writing – original draft, Writing – review & editing. XL: Data curation, Methodology, Writing – original draft, Writing – review & editing. YD: Data curation, Methodology, Supervision, Writing – original draft, Writing – review & editing. YS: Writing – original draft, Writing – review & editing, Formal analysis, Project administration. WH: Writing – original draft, Writing – review & editing, Formal analysis, Project administration, Validation. ZR: Writing – original draft, Writing – review & editing, Data curation, Methodology, Supervision. KD: Writing – original draft, Writing – review & editing, Data curation, Formal analysis, Methodology, Project administration. XZ: Conceptualization, Data curation, Formal analysis, Funding acquisition, Investigation, Methodology, Project administration, Resources, Software, Supervision, Validation, Visualization, Writing – original draft. WY: Conceptualization, Data curation, Formal analysis, Funding acquisition, Investigation, Methodology, Project administration, Resources, Software, Supervision, Validation, Visualization, Writing – original draft, Writing – review & editing.
