# Supplementary material for: Decoding Wilson disease: a machine learning approach to predict neurological symptoms
Source: Front Neurol. 2024 Jun 19;15:1418474. doi: 10.3389/fneur.2024.1418474 (PMC11223572; doi:10.3389/fneur.2024.1418474)
Supplement: Supplementary file 2 [file Data_Sheet_2.docx]

**Supplementary Materials**

**1. Details of classifiers**

**Logistic regression**

Logistic regression is a statistical model used for binary classification tasks, where the goal is to predict the probability that an instance belongs to a particular class[1]. Unlike linear regression, which predicts a continuous value, logistic regression predicts the probability of the instance belonging to the default class (class 1 in binary classification) using a logistic function.

The logistic function, also known as the sigmoid function, is used to map any real-valued number into a value between 0 and 1. This is essential for representing probabilities, as probabilities must fall within this range. The formula for the logistic function is:

$$P(y=1|x) =\frac{1}{1+e^{-z}}$$

Where$( P(y=1|x)$ is the probability that the instance belongs to class 1 given input $x$, and z is a linear combination of the input features $x$ and model parameters $\theta$:

$z = \theta_{0} + \theta_{1}x_{1} + \theta_{2} x_{2} + .... + \theta_{n}x_{n}$ \]

Here, $\theta_{0},\theta_{1},\theta_{2}.....\theta_{n}$are the model parameters (coefficients) that are learned during the training process, and $x_{1}{,x}_{2}.....x_{n}$ are the input features of the instance.

During the training process, the model learns the optimal values of the parameters $\theta$ that minimize a cost function, typically the cross-entropy loss function. Once the model is trained, it can be used to predict the probability that a new instance belongs to class 1 based on its features.

Logistic regression is a simple yet powerful algorithm that is widely used in various applications, including medical diagnosis, spam detection, and credit scoring, among others.

**Back propagation (BP) neural network**

Back propagation (BP) neural network[2], often simply referred to as neural network, is a type of artificial neural network that is widely used for supervised learning tasks, including classification and regression. It is composed of multiple layers of interconnected nodes, or neurons, organized in a hierarchical manner.

The basic structure of a BP neural network consists of three types of layers: Input Layer, Hidden Layers and Output Layer. The key concept behind BP neural network is the back propagation algorithm, which is used to train the network. The back propagation algorithm consists of two main steps: forward propagation and backward propagation.

BP neural network has several advantages, including their ability to learn complex non-linear relationships in data, their flexibility in handling different types of input and output data, and their scalability to large datasets. However, it also has some limitations, such as being prone to overfitting, the need for large amounts of training data, and the requirement for careful tuning of hyperparameters. Overall, BP neural network is a powerful and versatile tool for a wide range of machine learning tasks and have been successfully applied in areas such as image recognition, natural language processing, and financial forecasting.

**2. Supplementary Tables**

**Table S1.** The ranges of different hyperparameters used in grid search for classifiers.

| **Classifiers** | **C** | **Gamma** | **N_estimators** | | **Max_depth** | **Learning_rate** |
| --- | --- | --- | --- | --- | --- | --- |
| SVM | [0.5,1,2,4] | [1e-7,1e-6,1e-5,1e-4,  1e-3,1e-2,1e-1] | |  |  |  |
| RF |  |  | | [100,200,400,1000] | [2-8] |  |
| XGB |  |  | | [100,200,400,1000] | [2-8] | [0.05,0.01,0.5,0.2,0.1] |

**Table S2.** The best hyperparameter combinations for different species based on AUROC values obtained from the grid search.

| **Classifiers** | **C** | **Gamma** | **N_estimators** | | **Max_depth** | **Learning_rate** |
| --- | --- | --- | --- | --- | --- | --- |
| SVM | 2 | 1e-4 | |  |  |  |
| RF |  |  | | 400 | 4 |  |
| XGB |  |  | | 1000 | 4 | 0.1 |

**Table S3.** The hyperparameters used in BP and LR.

| **Classifiers** | **Learning_rate** | **Loss_funcation** | **epochs** | **hiden dim** | **out dim** |
| --- | --- | --- | --- | --- | --- |
| BP | 0.01 | CrossEntropyLoss | 100 | 64,32 | 1 |
| LR | 0.01 | BCELoss | 100 | - | 2 |

**3. Supplementary Figure**

**
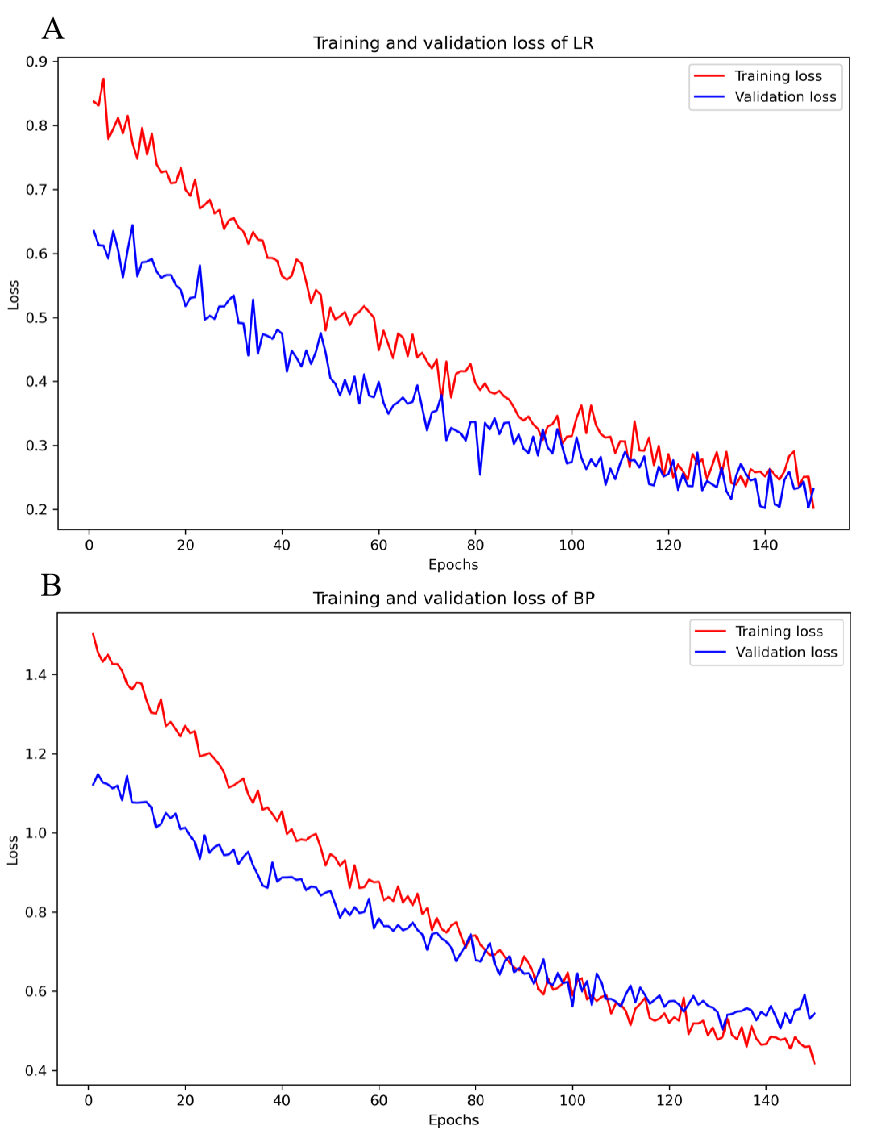
**

**Figure S1.** The loss curve of LR and BP. A, The loss curve of LR; B, The loss curve of LR.

**References**

[1] A. Das, Logistic regression, Encyclopedia of Quality of Life and Well-Being Research, Springer, 2024, pp. 3985-3986.

[2] X. Li, J. Wang, C.J.N.C. Yang, and Applications, Risk prediction in financial management of listed companies based on optimized BP neural network under digital economy. 35 (2023) 2045-2058.
